# Variations of Peroneus Tertius Muscle in Omani Population: A Surface Anatomical Study

**DOI:** 10.4314/ejhs.v31i5.19

**Published:** 2021-09

**Authors:** Srinivasa Rao Sirasanagandla, Al Wad Al Balushi

**Affiliations:** 1 Department of Human and Clinical Anatomy, College of Medicine and Health Sciences, Sultan Qaboos University, Muscat, Al-Khoudh 123, Oman; 2 College of Medicine and Health Sciences, Sultan Qaboos University, Muscat, Al-Khoudh 123, Oma

**Keywords:** Anatomical variation, foot, peroneus tertius muscle, surface anatomy

## Abstract

**Background:**

Peroneus tertius muscle (PTM) is a muscle of the anterior compartment of the leg. It is a highly variable muscle with a prevalence rate ranging from 42%-100% in different populations worldwide. We sought to explore the frequency of PTM and its existing variants, based on surface anatomical evaluation of the foot, in Omani subjects.

**Methods:**

In this study, a total of 222 adult Omani subjects (total 444 feet) were examined for the presence of PTM. The presence of PTM and its morphological types were identified based on surface anatomical examination using a standard palpation method. Descriptive statistics were used to present the data. The gender influence on the occurrence of PTM was determined by the Chi-square test.

**Results:**

The frequency of PTM was observed in 59.9% of total feet. A statistically significant gender difference with male dominance was observed on both right (p = 0.02) and left (p < 0.01) feet. Regarding PTM types, the F2 type was most common on both right (38.5%) and left feet (46.2%). The bilateral occurrence of PTM was found in 47.7% of cases.

**Conclusion:**

The frequency of PTM in Omani subjects is considerably high when compared to other Arab populations. The baseline information on the PTM proportion and distribution of its types is clinically important for physiotherapists and orthopedic surgeons.

## Introduction

Peroneus tertius muscle (PTM) or fibularis tertius muscle is a muscle of the anterior compartment of the leg. Its fibers originate from the medial distal third of the shaft of the fibula, the neighboring anterior part of the interosseous membrane, and the anterior crural intermuscular septum. Then, it runs anterior to the medial part of the lateral malleolus and is finally inserted into the foot. PTM is located lateral to the extensor digitorum longus muscle (EDLM) and has a strong association with this muscle. Due to the association, PTM is sometimes called the fifth tendon of EDLM (1–3). PTM causes dorsiflexion and eversion of the foot and evades hyper-inversion. In addition, it plays a role in the swing phase of walking by working to maintain the stability of the foot. Also, it prevents the toes from touching the ground by keeping them straight up (1–3). The size of the muscle and thickness of the tendon differs between two individuals (4). The muscle may have a double tendon. It is highly variable in its insertion. It may be inserted into the shaft or base of the fourth metatarsal bone, the shaft or base of the fifth metatarsal bone, or the fourth interosseous space. It may have an additional slip attached to the fourth metatarsal bone. It may give a tendinous slip to the extensor digitorum longus tendon to the little toe. In a few cases, PTM may have additional muscle belly called PTM accessories. All these variations were well explained by Bergmann et al (5).

PTM appears to be in three different forms in humans which are classified according to its belly and tendon association with EDLM. Every type can be easily differentiated by the surface anatomy of the muscle. The absence of the muscle is also recognizable (6,7). Many studies have estimated the prevalence of PTM in different populations worldwide with an ethnic variability (6–13). All these studies were either cadaver-based studies or surface anatomical studies. The reported PTM prevalence varied between 10% to 100%. The highest prevalence of PTM was reported in Bolivians (100%) (11). A recent clinical study has demonstrated a significant correlation between PTM presence and ankle injuries (8), though other previous studies did not support such association (13,14). PTM is associated with foot problems such as Jones fractures, tenosynovitis, and stress fractures (4,15,16). The insertion of the PTM tendon into the fifth metatarsal bone exhibit more risk to have avulsion fracture particularly at the tuberosity of the fifth metatarsal bone (15,16). On the other side, it is considered as an accessory muscle of eversion and dorsiflexion of the foot, and its absence does not affect these functions of the foot (1–3). Hence, it has a remarkable clinical use as a muscle flap to fill the soft tissue defects in the foot (17). Its muscle flap along with tendon is frequently used to correct ankle joint laxity and in foot drop transplantation surgeries (18). PTM plays an important role over ankle arthroscopy where it marks the anterolateral border for the procedure (4). Knowledge of anatomical variants of PTM is important for clinicians particularly orthopedic surgeons and foot surgeons (19). Despite the increasing clinical significance and varied anatomy of PTM, its prevalence has not been explored across populations worldwide. Further, to date, there is no study conducted on the Omani population. Therefore, we sought to explore the frequency of PTM muscle, based on surface anatomical evaluation of foot, in (SQU) Omani subjects.

## Methods

This is an observational cross-sectional study conducted at Sultan Qaboos University (SQU), located in Muscat, Oman. SQU Omani subjects of both genders were included in the study. Subjects who have a recent foot injury, lower limb paralysis, or congenital foot anomalies along with non-Omani subjects were excluded from the study. The data were collected for about six months between 1st October 2019 and 31st March 2020.

Institutional ethical committee approval for this study was obtained (SQU-EC/159/19) from the Medical Research Ethics Committee, College of Medicine and Health Sciences, Sultan Qaboos University.

**Figure 1:**
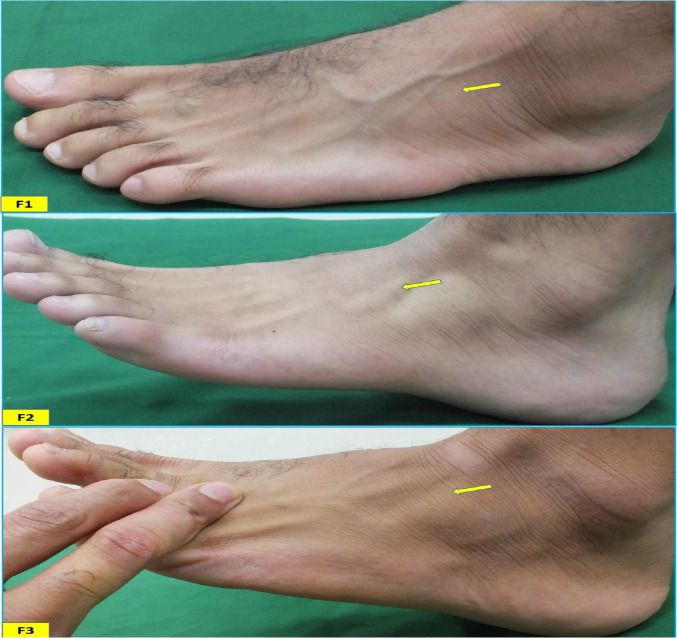
Dorsolateral view of the left foot and ankle showing the tendon of the peroneus tertius (arrow) observed in F1, F2 and F3

**Data collection**: The subjects were examined according to the protocols described by Tixa (20), Kendall et al. (21), and Ramirez et al. (22). Using their protocols, a series of pilot studies were conducted for accurate identification of the PTM. In the present study, each subject was asked to sit with a flexed knee (about 110°) and a supported ankle. The process of identification was performed in three steps: The first step: the tendon was examined by palpation toward its insertion gently, without muscular tension. If the tendon was recognized; it was noted as an F1 type (Figure 1). If not; moved to the second step. The second step: the subject was asked to evert and dorsiflex each foot. If the tendon was recognized on the same course of the first step, it was noted as an F2 type (Figure 1). If not, moved to the third step. The third step: the subject was asked to evert and dorsiflex the foot with resistance applied among the lateral border of the foot by evaluators' hands. If the tendon was visualized or palpated, it was noted as an F3 type (Figure 1). If not observed, PTM was considered as absent.

**Statistical analysis**: Statistical Package for Social Sciences version 23 (SPSS) was used for the data analysis. Descriptive statistics were used to present the data of the PTM frequency and its types. A Chi-square test was used to determine the association between gender and the frequency of PTM. P < 0.05 was considered as significant.

## Results

**Figure 2:**
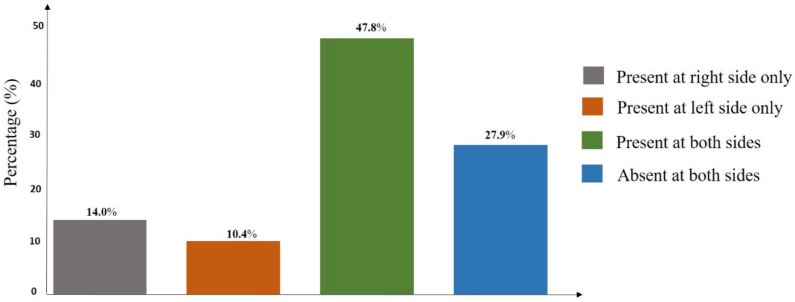
The distribution of peroneus tertius muscle among Omani subjects

**Figure 3:**
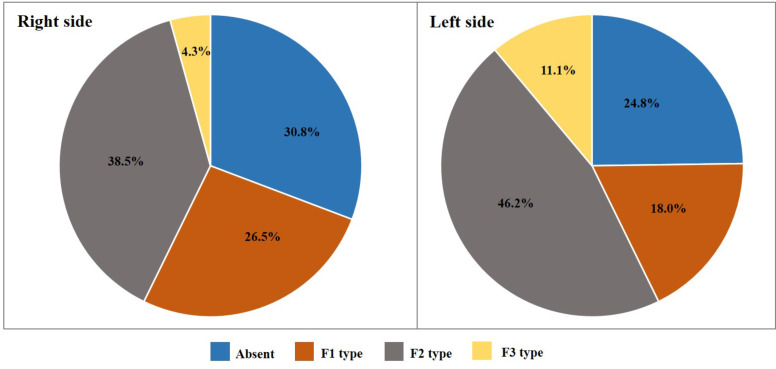
The frequency of each type of peroneus tertius muscle (F1, F2, and F3) in males

**Figure 4:**
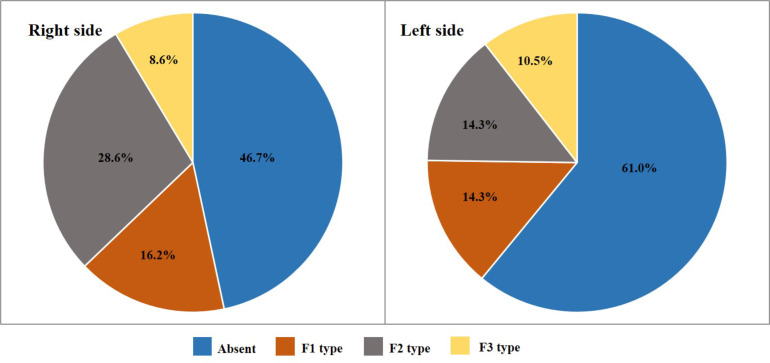
The frequency of each type of peroneus tertius muscle (F1, F2, and F3) in females

A total of 222 (117 males and 105 females) (SQU) Omani subjects with informed consent were included in the study. The average age of the sample was 20.2 ± 1.45 years. Out of 444 feet studied, 61.7% and 58.1% of PTM were observed, in the right feet and left feet respectively with an overall frequency of 59.9% (266/444). The gender-wise distribution of the overall presence of PTM on right and left feet was presented in Table 1. The frequency of presence of PTM was significantly more in males on both right (p = 0.02) and left (p < 0.01) feet. The percentage distribution of the symmetry of PTM among 222 participants was presented in Figure 2. Concerning the presence of PTM, bilateral symmetry was found in 47.8% of total cases. In males and females, the distribution of each type of PTM (Figures 1, 2, and 3) on both right and left feet was presented in Figure 3 and Figure 4, respectively. On the right side, the F2 type was the most common type with similar percentages in both males (38.5%) and females (28.6%). F1 type was the second most common type in both males (26.5%) and females (16.2%) with a little difference in frequency. F3 type was the least common type in both males (4.3%) and females (8.6%). On the left side, the F2 type was the most common in males (46.2%) followed by the F1 type (18.0%) and then the F3 type (11.1%). In females, both F1 and F2 types showed a similar frequency in distribution with a percentage of 14.3%. F3 was the least common type with a percentage of 10.5%.

**Table 1:** Association between prevalence of PTM and gender

	Right side* (n=222)		Left side# (n=222)	
**Gender**	Male (n=117)	Female (n=105)	Male (n=117)	Female (n=105)
**Number**	81	56	88	41
**Prevalence of PTM (%)**	69.2%	53.3%	75.2%	39.0%

Significant difference with respect to gender in the right (* p = 0.02) and left (# p< 0.01) feet

## Discussion

The present study is the first to report the frequency of PTM and its existing variants in Omani subjects. In Omani subjects, the frequency of PTM was found to be 59.9%. The reported prevalence of PTM worldwide varied greatly among different populations (Table 2). In far east countries, it was varied between 89.3% to 95.5% (4). A high frequency of PTM was observed in the western population (97.4%) (12). In Indians and Africans, it was found to be 52% and 90.2%, respectively (10,13). A recent study explored the PTM prevalence among five different Arab populations. Three out of these five populations were from the gulf region; Saudi, Bahraini, and Kuwaiti (8). This study results demonstrated the PTM prevalence of 38.5%, 42%, and 41.2% in these populations, respectively. In the same study prevalence of PTM in North African populations (Tunis and Egypt) was found to be 67.7% and 42.8%, respectively (8). A high frequency of 95.4% PTM was observed in a study from Turkey (16). The frequency of PTM reported in the present study is higher than that reported in other Arab populations of Bahrain, Saudi, and Kuwait. However, it is close to the frequency reported in the Indian population of 52%. The wide discrepancy in the prevalence between the populations could be due to genetic factors. The genetic variations are indicated by ethnic background and geographical differences.

In the present study higher frequency of PTM was observed in the right feet. Similar results were observed in Tunisian, Egyptian, Saudi, and Kuwaiti subjects (8). Contrary to this, in Bahraini and Indian subjects it was more on left feet (8,10). Furthermore, similar to the present study of Omani subjects, in Arab, north African, Indian, and Nigerian subjects a high frequency of bilateral presence of PTM was reported (8,10,13).

The majority of the previous studies have confirmed the sexual dimorphism in the occurrence of PTM with male dominance (4). A high frequency of PTM in males was observed in Saudi and Egyptian, Indian, Belgian and Chilean populations (8,10,14,22). Similar to these studies, the current study also revealed a high PTM occurrence in males. However, in Bahraini, Kuwaiti, and Tunisian populations it was found to be more in females (8). Further research on a large sample is required to confirm the sexual dimorphism.

In the current study, concerning the distribution of PTM types, the F2 type was the most common type on both right and left feet (38.5%, 46.2%). Similar results were observed in Bahraini (39%, 48.9%), and Kuwaiti (54.5%, 47.2%) populations with predominant F2 type (8). Contrary to these results, In Egyptians, Indian and Nigerian populations F3 type was the most common type of PTM (8,10,13). The discrepancy in the morphological features of PTM observed between these populations could be explained based on the complex development of common muscle mass that gives rise to both EDL and PTM (31). The PTM shows a wide variation in its morphology particularly in the attachments (32,33). The complete absence of the PTM on both sides was the most common variation that was frequently reported by various authors (5). Morphological variations of PTM could also be of evolutionary significance (4). PTM was not observed in new world monkeys and *Strepsirrhini* (4) but rarely reported in the pig-tailed baboon *(Papio ursinus)* (34) and the Toque monkey *(Macaca Sinica)* (35). In Crab-eating monkeys *(Macaca fascicularis)* it was found in 2.9% of male specimens (36). A higher frequency of PTM was noted in great apes. It was noted with a frequency of 5% in *chimpanzees* (37) and 29.6% in *gorillas* (36). The differences in PTM frequencies among arboreal apes and terrestrial apes indicate that evolutionary acquisition of PTM is associated with bipedalism (4).

Observations of anatomical variations deepen existing knowledge and can also be useful for clinicians in their daily practice (17). PTM is frequently used to reconstruct soft tissue defects in the lower limb (38). It is used as a local muscle flap for the treatment of osteomyelitis, in the lower limb (38). Its tendon is preferred in surgical procedures such as resection of the foot, tendoplasty, tendon transfer, and ligament reconstructions particularly ankle joint (39). A recent study has demonstrated a hypertrophied tendon and/or low-lying muscle belly of PTM causing anterolateral ankle and rearfoot pain (40). Hence, prior knowledge of the frequency and anatomical variants of PTM is clinically relevant for orthopedic surgery and physiotherapeutic view.

A limitation of the current study is that through the surface anatomy examination, the PTM identification may be missed in a few cases. Using a gold standard imaging method in identifying PTM will be the strong study design. Hence, the analysis of the PTM morphology using imaging techniques to understand its role in the biomechanics of ankle injuries and foot pain is warranted.

In conclusion, the present study documented for the first time the frequency of PTM in the Omani subjects. The frequency of PTM in Omani subjects is considerably high when compared to other Arab populations. The baseline information on the PTM frequency and distribution of its types is clinically important for physiotherapists and orthopedic surgeons.
